# A retrospective analysis of disease epidemiology, comorbidities, treatment patterns, and healthcare resource utilization of alopecia areata in the United Arab Emirates using claims database

**DOI:** 10.1111/1346-8138.17381

**Published:** 2024-07-25

**Authors:** Anwar Al Hammadi, Nisha V. Parmar, Mohamed Farghaly, Sara Al Dallal, Mostafa Wagdy Abdullah Zayed, Fadwa Ebeid, Kumaresan Subramanyam, Badarinath Chickballapur Ramachandrachar, Haytham Mohamed Ahmed

**Affiliations:** ^1^ DermaMed Clinic Dubai UAE; ^2^ Dubai Health Authority Dubai UAE; ^3^ Pfizer Inc. Ltd Dubai UAE; ^4^ EMEA Consulting Services IQVIA Bengaluru India; ^5^ Real‐World Evidence, IQVIA Dubai UAE

**Keywords:** alopecia areata, autoimmune disease, dermatologist, healthcare costs, United Arab Emirates

## Abstract

Alopecia areata (AA) is an autoimmune disorder that manifests as nonscarring hair loss and imposes a substantial disease burden. The current study, using an e‐claims database, assesses the disease burden, comorbidities, treatment patterns, specialties involved in the diagnosis of AA, healthcare resource utilization (HCRU), and associated costs in privately insured patients with AA in Dubai, United Arab Emirates. The retrospective longitudinal secondary study was conducted using Dubai Real‐World Database e‐claims data during 01 January 2014 to 30 June 2022. Patients with at least one diagnosis claim of AA during the index period (01 January 2015–30 June 2021) with continuous enrollment (one or more AA/non‐AA claim in the post‐index period) were included in the analysis. The patients were stratified into subcohorts based on diagnosis code and treatment patterns, as mild, **moderate‐to‐severe**, and others. Demographics, comorbidities, treatment patterns, specialists visited, and HCRU were assessed. The study included 11 851 patients with AA (mean age: mild: 37 years; **moderate‐to‐severe**: 36 years), with a male predominance (mild: 77.6%; **moderate‐to‐severe**: 60.8%). The most prevalent comorbidities in the moderate‐to‐severe AA subcohort were autoimmune and T‐helper 2–mediated immune disorders, including contact dermatitis and eczema (62.1%), atopic dermatitis (36.1%), and asthma (36.1%). Most patients consulted dermatologists for treatment advice (mild AA: 87.4%; moderate‐to‐severe AA: 47.7%) and, notably, within 1 day of AA diagnosis. Topical steroids were frequently prescribed across cohorts, regardless of disease severity. Analysis of comorbidities among patients with AA indicated an additional HCRU burden among these subsets of patients. The median disease‐specific HCRU cost was higher for psychological comorbidities versus autoimmune and T‐helper 2–mediated immune disorders (US $224.99 vs US $103.70). There is a substantial disease and economic burden in patients with AA and associated comorbid conditions; therefore, investing in novel therapies that target the underlying autoimmune pathway may address the gap in effective management of AA.

## 1 INTRODUCTION

Alopecia areata (AA) is an autoimmune disorder characterized by deep tissue inflammation leading to impairment of the function of hair follicles and subsequent nonscarring hair loss that has a substantial impact on the quality of life (QOL) of affected patients.^1^, ^2^, ^3^ AA typically manifests as well‐demarcated patches of hair loss on the scalp and other hair‐growing regions with a sudden onset and may progress to involve the entire scalp (alopecia totalis) or the whole body (alopecia universalis).^2^ AA affects individuals of all ages and sexes, with an estimated lifetime prevalence of 2% globally.^1^, ^2^ According to the Global Burden of Disease (GBD) 2019, the overall prevalence of AA was 18.4 million worldwide, with a female preponderance.^4^ The age‐standardized prevalence and incidence rates, as reported in the systemic analysis of GBD between 1990 and 2019, were twofold higher in females than in males, with ages in the range of 30 to 34 years.^5^ The overall prevalence of AA in Saudi Arabia was found to be 2.3%, with a mean onset age of 25.6 years.^6^ The disability‐adjusted life‐years for patients with AA were 0.601 million, with the rate of AA being 7.51 per 100 000 and a higher disability associated with female patients.^4^, ^5^

The exact pathophysiology of AA is not clearly defined; however, an autoimmune cause with genetic propensity is considered to play a crucial role in the occurrence of disease and remains the most widely accepted hypothesis. Epigenetic factors (environment and stress) and circulating autoantibodies and autoantigens in the affected regions also play a key role in the autoimmune process.^2^ Previous studies have reported an association between AA and several autoimmune diseases, including autoimmune thyroid disease, vitiligo, systemic lupus erythematosus, and scleroderma.^2^ Other comorbidities prevalent in patients with AA include rheumatoid arthritis, celiac disease, atopic diseases, psoriasis, psychological disorders, gastrointestinal diseases, nutritional deficiencies, ocular abnormalities, and diabetes.^6^, ^7^, ^8^ Hence, it is important to effectively manage comorbid conditions to achieve better clinical outcomes in patients with AA.

Furthermore, the psychological and emotional well‐being of patients is negatively compromised in AA. In addition to anxiety and depression, patients experience a decrease in overall health‐related QOL in areas such as personality, social functioning, emotions, behaviors, vitality, and mental health.^9^, ^10^, ^11^, ^12^ In studies conducted using Dermatology Life Quality Index (DLQI) questionnaires, the mean scores obtained for severe and mild AA (10.7 ± 7.5 and 5.4 ± 6.8, respectively) indicated impaired QOL.^11^

The primary goals in the management of AA include restoration and maintenance of hair growth.^13^ However, traditional treatment modalities, including corticosteroids (topical, intralesional, and systemic), contact immunotherapy (squaric acid dibutylester or diphenylcyclopropenone), minoxidil, anthralin, cyclosporine, methotrexate, and phototherapy, have limited evidence and may fail to achieve the treatment goal due to varied treatment responses, frequent relapses, and varied adverse effect profiles.^14^, ^15^, ^16^

Recent insights and a comprehensive understanding of the pathogenesis of AA have led to the development of novel therapies for AA.^14^ The effectiveness of Janus kinase (JAK) inhibitors in the management of AA has been highlighted in recent studies. JAK inhibitors simultaneously target multiple pathways and have proven anti‐inflammatory properties and thereby resolve inflammation, restore normal hair follicle function, and promote hair growth.^14^, ^17^, ^18^, ^19^, ^20^, ^21^ As a result of these studies, the United States, Europe, and Japan have approved baricitinib in adults and more recently ritlecitinib in adolescents and adults for the treatment of severe AA. Apart from pharmacological interventions, psychotherapeutic interventions and psychoeducation constitute important aspects of AA management.^22^

In addition to the disease and psychological burden, AA also imposes a substantial economic burden. Out‐of‐pocket (OOP) expenses are considerably high in AA, accounting for copayments, partial insurance coverage, hair appointments, nutritional supplements, and alternative therapies.^23^ The annual median OOP spending of patients in an American study was observed to be $1354.^23^ In a retrospective analysis, the total all‐cause cost ($18 988 vs $11 030) and OOP expense ($2685 vs $1457) were higher in patients with AA versus controls.^24^ The disease‐specific cost was estimated at $419.12, attributed to outpatient and prescription costs.^25^ In addition, increased healthcare resource utilization (HCRU) was observed in patients with AA; emergency visits, ambulatory visits, and pharmacy visits were the key drivers of HCRU^26^, ^27^ A major challenge in the management of AA is the denial of insurance coverage, considering the disease as a cosmetic issue, thereby leading to significant OOP expenses.^28^, ^29^

Despite the substantial disease and psychosocial burden and gaps in management for patients with AA in the United Arab Emirates (UAE) region, there is a paucity of real‐world data (lack of established registries and robust epidemiological data) on patient characteristics, treatment patterns, comorbidities, and HCRU for AA. These data are crucial in assisting policymakers and healthcare professionals in formulating optimal guidelines and bridging knowledge gaps. Therefore, the current study, using an e‐claims database, was conducted to assess the disease burden, comorbidities, treatment patterns, and specialties involved in the diagnosis of AA, as well as to assess HCRU in patients with AA in Dubai, UAE.

## 2 METHODOLOGY

### 2.1 Study design

**FIGURE 1:**
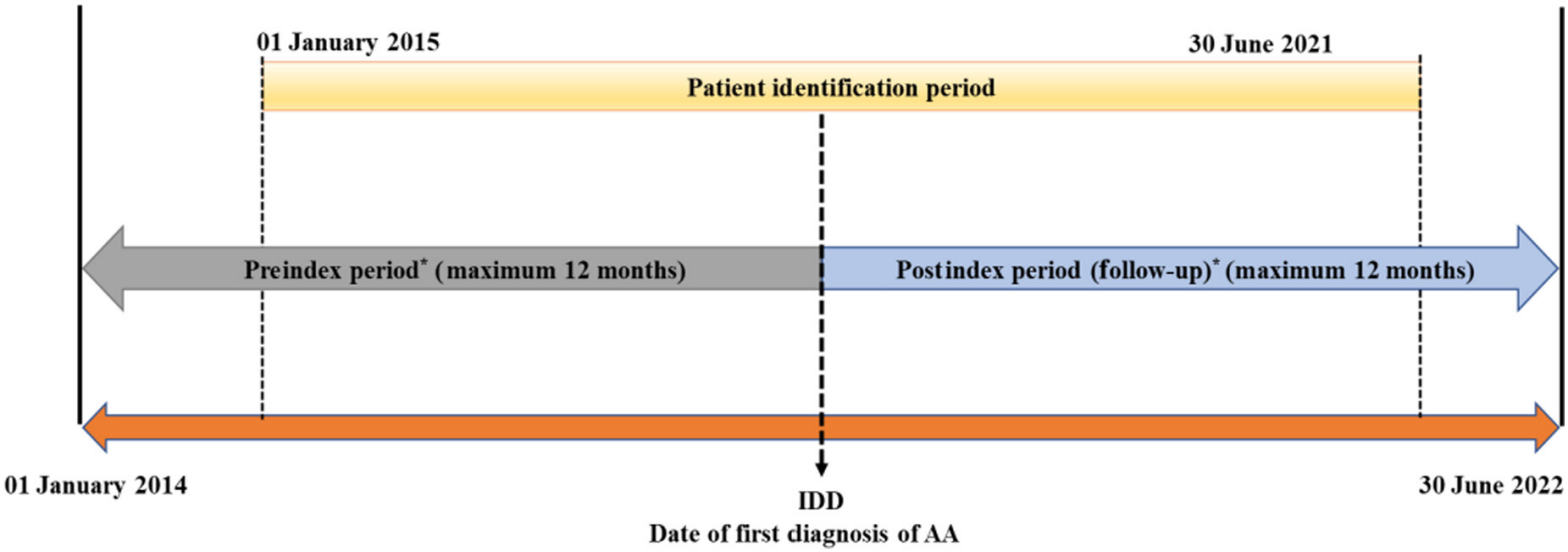
Overview of study design. *Pre‐index and post‐index periods varied for each patient based on the index diagnosis date (IDD). Patient with at least one claim (alopecia areata [AA]/non‐AA) at any time during the 12‐month post‐index period (follow‐up) (continuous enrollment).

This retrospective longitudinal secondary study was conducted using Dubai Real‐World Database (DRWD) e‐claims data during 01 January 2014 to 30 June 2022. The date of the first diagnosis of AA during the index period or patient identification period (01 January 2015–30 June 2021) was termed the index diagnosis date (IDD). The period starting from the IDD until 12‐month follow‐up was considered the post‐index period (Figure 1).

### 2.2 Data sources

The DRWD e‐claims database is an anonymous, longitudinal patient‐level database comprising insurance‐related claims for approximately 100% of the population covered by private insurance. (In Dubai, the public healthcare system is primarily used by Emiratis while the expatriates who make up to 85% of the total population are privately insured). This population predominantly represents the multiethnic expat population (89%).^30^ The claims database contains complete information pertaining to patients' demographics, diagnosis, procedures (medical, surgical, and diagnostic), prescriptions, other related services, treatment, and consultations.

### 2.3 Study population

The patients were identified using *International Classification of Diseases, Tenth Revision, Clinical Modification* (*ICD‐10‐CM*) codes. The study included patients with at least one diagnosis claim of AA anytime during the index period with continuous enrollment (one claim [AA/non‐AA] in the post‐index period). Patients with the diagnosis of trichotillomania, androgenic alopecia, telogen effluvium, tinea capitis, tinea barbae, scarring alopecia, unspecified nonscarring hair loss, pseudopelade, and folliculitis decalvans were excluded from the study.

The patients included in the study were further stratified into subcohorts based on their diagnosis and selected prescription medicines, following the diagnosis of AA. The subcohorts are described as: (i) mild AA: patients with an AA diagnosis code for other AA (L63.8) or AA, unspecified (L63.9), and those who were prescribed topical nonsteroids (minoxidil, pimecrolimus, tacrolimus), topical steroids (hydrocortisone acetate, mometasone furoate, clobetasol propionate, hydrocortisone, neomycin sulphate, gramicidin, nystatin, triamcinolone acetonide, miconazole nitrate, prednicarbate, clotrimazole, betamethasone valerate, allantoin, coal tar, hydrocortisone, clioquinol+hydrocortisone acetate, fluocinonide/gentamicin sulphate, hydrocortisone 17‐butyrate, crotamiton/hydrocortisone), intralesional triamcinolone, nontraditional treatments, or other treatments were categorized as having mild disease; (ii) moderate‐to‐severe AA: patients with a diagnosis code for alopecia universalis (L63.0) or alopecia totalis (L63.1) or ophiasis (L63.2) and patients with a diagnosis code for other AA (L63.8) or AA, unspecified (L63.9), and those who were prescribed any immunomodulators (cyclosporine, methotrexate, ruxolitinib, azathioprine, ixekizumab, sulfasalazine, JAK inhibitors, dupilumab, apremilast), oral steroids (dexamethasone, prednisolone, betamethasone, fludrocortisone acetate, cortisone acetate), systemic nonsteroids (dapsone), finasteride, systemic antihistamines (only fexofenadine and ebastine) phototherapy, or platelet‐rich plasma; and (iii) others: patients with a diagnosis code for AA (L63) and not captured in the mild and moderate‐to‐severe disease cohorts.

### 2.4 Ethical considerations

Anonymized structured data were used for the current observational study that did not involve any intervention. Hence, patient informed consent was not a requisite. The approval of an institutional review board to conduct this study was not obligatory, since it did not involve the collection, use, or transmission of individually identifiable data. The study was conducted in accordance with the Health Insurance Portability and Accountability Act of 1996, thereby preventing the disclosure of patient health information.

### 2.5 Baseline variables and outcomes

Patient demographics, including age (latest available age at the time of data extraction), sex, insurance plan, and nationality, were evaluated during the index period (01 January 2015–30 June 2021) for mild, moderate‐to‐severe, and other AA–diagnosed subcohorts of patients.

Reported new visits and repeat visits of the overall study population were evaluated yearly during the index period (01 January 2015–30 June 2021).

Clinical characteristics and comorbidities were evaluated at the time of diagnosis of AA (index date) and for the entire patient journey, for the mild and moderate‐to‐severe AA–diagnosed subcohorts of patients. Patients were classified into three comorbidity groups namely autoimmune and T‐helper 2 (Th2)–mediated immune disorders, psychological disorders, and other comorbidities.

Autoimmune and Th2‐mediated immune disorders included contact dermatitis and eczema, atopic dermatitis, asthma, ulcerative colitis, psoriasis, autoimmune thyroid disorder, rheumatoid arthritis, chronic urticaria, vitiligo, Crohn disease, celiac disease, hypersensitivity, systemic lupus erythematosus, Sjogren syndrome, uveitis, hidradenitis suppurativa, ankylosing spondylitis, reactive arthritis, multiple sclerosis, psoriatic arthritis, and systemic sclerosis. Psychological disorders included anxiety and depression. Other comorbidities included hyperlipidemia, thyroid disorder, diabetes, hypertension, osteoarthritis, coronary heart disease, obesity, cerebrovascular disease, tuberculosis, tobacco dependency, and alcohol abuse.

Treatment patterns, in terms of the percentage of patients on treatment (prescription of medications), were evaluated during the 12‐month post‐index period in the mild and moderate‐to‐severe AA–diagnosed subcohorts of patients. In addition, the average time between first AA diagnosis and initiation of treatment was calculated during the study, from the index date until the end of the study period and was evaluated in the mild and moderate‐to‐severe AA–diagnosed subcohorts of patients.

Frequency of visits by specialty type (e.g., dermatology, general practice, and pediatrics) was evaluated during the 12‐month post‐index period in the mild and moderate‐to‐severe AA–diagnosed subcohorts of patients.

The average time to visit a dermatologist from index date and first visit to dermatologist was evaluated in the overall study population.

The HCRU and associated costs (all‐cause and disease‐specific) by groups of comorbidities (autoimmune and Th2‐mediated immune disorders, psychological disorders, and other comorbidities) were assessed by visit type (inpatient visits, emergency department [ED] visits, and outpatient visits) and activity type (medications, Current Procedural Terminology [procedures], Healthcare Common Procedure Coding System [consumables including medical and surgical supplies], services, and DRG). DRG is a medical insurance payment standard. Cases are allocated to different groups in accordance with actual situations such as the complexity of the disease and the consumption of medical resources, which are determined by the main diagnosis of the disease and combined with comorbidities, surgery and treatment operations, and so on. It is a relatively scientific and advanced medical payment model recognized globally. The result was represented in the form of gross cost and net cost, respectively. Claims with a claim amount of less than 1 Emirati Dirhams were excluded from the analysis.

### 2.6 Data sources and measurement

The descriptive analysis was conducted with continuous variables summarized as mean, standard deviation, median, and minimum and maximum, as appropriate. Categorical variables are summarized by frequency and percentages.

All study variables, including demographic and clinical characteristics, reported new visits and repeat visits, treatment patterns, were evaluated for overall patients with AA and subcohorts of mild and moderate‐to‐severe AA–diagnosed patients. The HCRU and associated costs (all‐cause and disease‐specific) were analyzed for overall AA and subcohorts of patients with mild and moderate‐to‐severe AA, by groups of specific comorbidities.

## 3 RESULTS

### 3.1 Demographics of patients with AA

Of the 21 369 total patients, 11 851 were selected as the study population based on the inclusion criteria (Tables S1 and S2). The selected population was further stratified into three subcohorts based on disease severity: 2639 (22.3%) with mild AA, 1144 (9.7%) with moderate‐to‐severe AA, and 8068 (68.1%) as “others.”

The mean age of the patients across the subcohorts was in the range of 36 to 37 years, with most of the patients being older than 18 years (91.1%–91.4%). Overall, a higher proportion of males were affected by AA (60.8%–77.6%), with the maximum representation from the mild AA subcohort (*n* = 692, 77.6%). Most patients (70.0%–73.2%) opted for premium insurance plans. The common nationalities of patients with AA included Indian (37.8%–46.0%), Pakistani (9.9%–12.5%), Egyptian (7.1%–9.5%), British (2.3%–4.4%), and Jordanian (2.9%–3.8%) (Table 1).

**TABLE 1:** Patient demographic and clinical characteristics for the subcohort population.

Patients with mild AA	Patients with moderate‐to‐severe AA	Others
*N* (%)	*N* (%)	*N* (%)
Overall study population in the index time period	2639 (100)	1144 (100)	8068 (100)
Overall study population with demographics available	892 (33.8)	347 (30.3)	2476 (30.7)
Overall study population with demographics missing	1747 (66.2)	797 (69.7)	5592 (69.3)
Age (latest available age, in years)			
0–11	43 (4.8)	15 (4.3)	114 (4.6)
12–17	34 (3.8)	16 (4.6)	103 (4.2)
>18	815 (91.4)	316 (91.1)	2259 (91.2)
Age statistics, years			
Mean	37	36	36
Median	38	38	37
SD	11	12	11
Minimum	4	6	2
Maximum	75	76	76
25th percentile	33	32	32
75th percentile	44	44	43
IQR	11	12	11
Sex			
Male	692 (77.6)	211 (60.8)	1798 (72.6)
Female	200 (22.4)	136 (39.2)	677 (27.3)
Insurance plan			
Basic	241 (27.0)	93 (26.8)	742 (30.0)
Premium	651 (73.0)	254 (73.2)	1734 (70.0)
Nationality distribution			
India	337 (37.8)	158 (45.5)	1138 (46.0)
Pakistan	88 (9.9)	43 (12.4)	310 (12.5)
Egypt	85 (9.5)	26 (7.5)	175 (7.1)
Britain	39 (4.4)	11 (3.2)	58 (2.3)
Jordan	34 (3.8)	10 (2.9)	78 (3.2)
Philippines	28 (3.1)	14 (4.0)	130 (5.3)
Lebanon	28 (3.1)	1 (0.3)	44 (1.8)
Syria	27 (3.0)	7 (2.0)	52 (2.1)
Emirates	19 (2.1)	7 (2.0)	71 (2.9)
Nepal	16 (1.8)	5 (1.4)	43 (1.7)
Canada	14 (1.6)	5 (1.4)	18 (0.7)
Bangladesh	11 (1.2)	5 (1.4)	49 (2.0)
United States	11 (1.2)	5 (1.4)	22 (0.9)
Palestine	10 (1.1)	1 (0.3)	27 (1.1)
Comorbidities			
Overall study population with comorbidities	2371 (89.8)	988 (86.4)	
Autoimmune and Th2‐mediated immune disorders	2225 (84.3)	831 (72.6)	
Contact dermatitis and eczema	2096 (79.4)	710 (62.1)	
Atopic dermatitis	1226 (46.5)	413 (36.1)	
Asthma	328 (12.4)	118 (10.3)	
Ulcerative colitis	206 (7.8)	62 (5.4)	
Psoriasis	122 (4.6)	69 (6.0)	
Thyroid disorder—autoimmune	103 (3.9)	70 (6.1)	
Rheumatoid arthritis	72 (2.7)	61 (5.3)	
Chronic urticaria	41 (1.6)	24 (2.1)	
Vitiligo	29 (1.1)	9 (0.8)	
Systemic lupus erythematosus	22 (0.8)	32 (2.8)	
Psychological disorders	80 (3.0)	45 (3.9)	
Anxiety	70 (2.7)	38 (3.3)	
Depression	24 (0.9)	18 (1.6)	
Others	1242 (47.1)	642 (56.1)	
Hyperlipidemia	791 (30.0)	342 (29.9)	
Thyroid disorder	653 (24.7)	431 (37.7)	
Diabetes	424 (16.1)	201 (17.6)	
Hypertension	383 (14.5)	169 (14.8)	
Osteoarthritis	184 (7.0)	79 (6.9)	
Coronary heart disease	108 (4.1)	30 (2.6)	
Obesity	42 (1.6)	27 (2.4)	

*Note*: Comorbidities was assessed only in the mild alopecia areata (AA) and moderate‐to‐severe AA group and was not assessed in the “others” AA group.

Abbreviations: IQR, interquartile range; SD, standard deviation; Th2, T‐helper 2.

### 3.2 Clinical characteristics of patients with AA

Data pertaining to comorbidities during the study period were available for 2371 (89.8%) and 988 (86.4%) in the mild AA and moderate‐to‐severe subcohorts, respectively. Most patients were affected by autoimmune and Th2‐mediated immune disorders (mild AA: *n* = 2225, 84.3%; moderate‐to‐severe AA: *n* = 831, 72.6%), followed by other comorbidities (mild AA: *n* = 1242, 47.1%; moderate‐to‐severe AA: *n* = 642, 56.1%). The most prevalent autoimmune and Th2‐mediated immune disorders were contact dermatitis and eczema (mild AA: *n* = 2096, 79.4%; moderate‐to‐severe AA: *n* = 710, 62.1%), followed by atopic dermatitis (mild AA: *n* = 1226, 46.5%; moderate‐to‐severe AA: *n* = 413, 36.1%) and asthma (mild AA: *n* = 1226, 46.5%; moderate‐to‐severe AA: *n* = 413, 36.1%). In the other comorbidities category, the most frequently occurring comorbidity was hyperlipidemia (mild AA: *n* = 791, 30.0%; moderate‐to‐severe AA: *n* = 342, 29.9%), thyroid disorder (mild AA: *n* = 653, 24.7%; moderate‐to‐severe AA: *n* = 431, 37.7%), and diabetes (mild AA: *n* = 424, 16.1%; moderate‐to‐severe AA: *n* = 201, 17.6%). Anxiety (mild AA: *n* = 70, 2.7%; moderate‐to‐severe AA: *n* = 38, 3.3%) and depression (mild AA: *n* = 24, 0.9%; moderate‐to‐severe AA: *n* = 18, 1.6%) were the commonly occurring psychological disorders in patients with AA (Table 1).

### 3.3 Reported new visits and repeat visits of patients with AA

**FIGURE 2:**
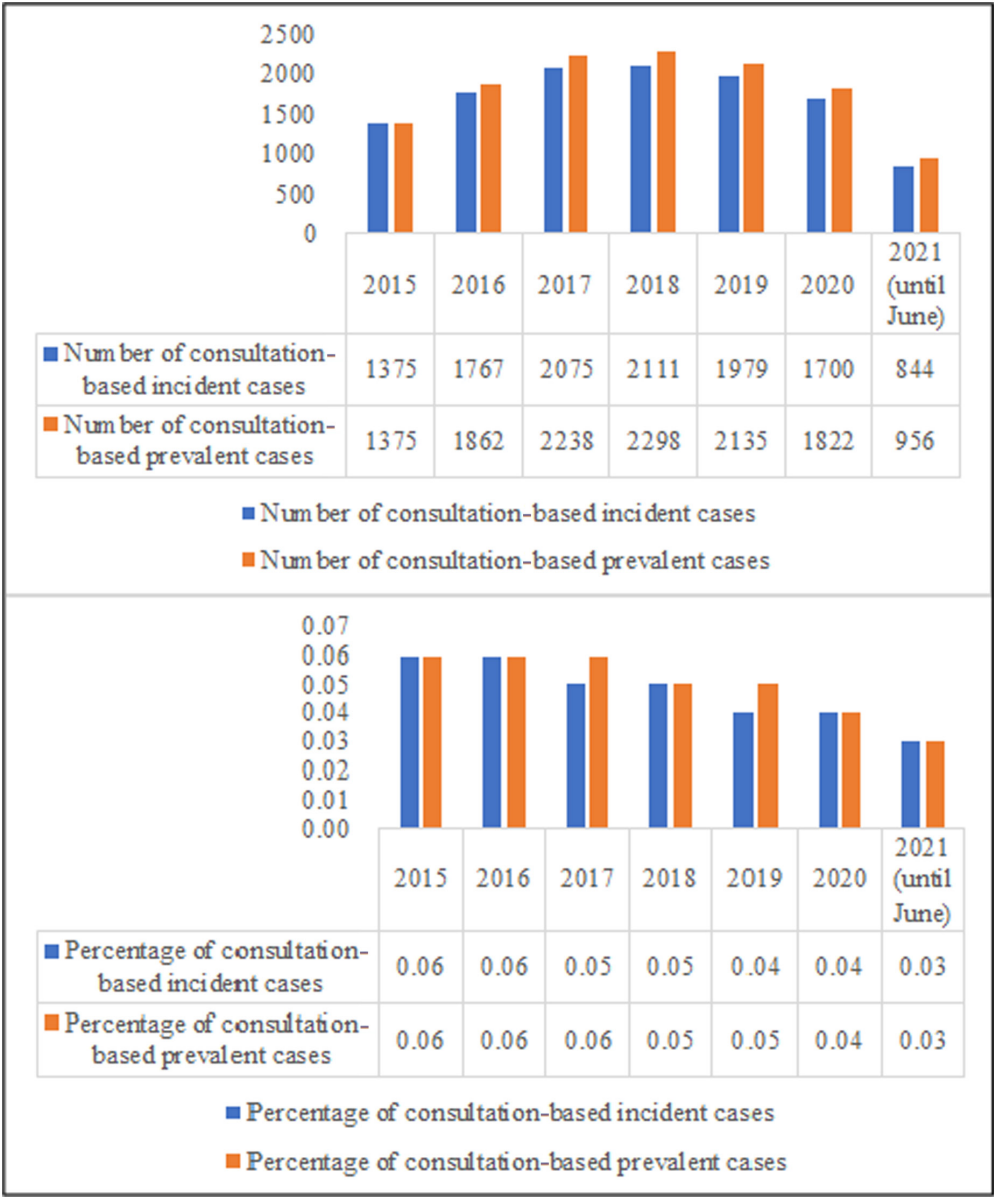
Comparison of number and percentage of new visits and repeat visits in patients with alopecia areata.

A steady decrease, both in the percentages of reported new visits and repeat visits, was observed from 0.06% in 2015 to 0.03% in 2021 (until June) (Figure 2).

### 3.4 Treatment patterns

Data related to the treatment pattern from the index date to 12‐month follow‐up period were available for 2432 patients (92.2%) in the mild AA subcohort and 537 patients (46.9%) in the moderate‐to‐severe AA subcohort. The most prescribed treatment in the mild AA subcohort was topical steroids (*n* = 1902, 72.1%), topical nonsteroids (*n* = 552, 20.9%), and intralesional triamcinolone (*n* = 391, 14.8%). Patients in the moderate‐to‐severe subcohort were mostly prescribed topical steroids (*n* = 309, 27.0%), oral steroids (*n* = 260, 22.7%), and systemic antihistamines (*n* = 158, 13.8%) (Table 2).

**TABLE 2:** Treatment pattern for patients with AA.

	Treatment prescription patterns (from index date to the 12‐month post‐index period), *N* (%)
*Patients with mild AA*	
Overall study population	2639 (100)
Overall study population with treatment	2432 (92.2)
Topical steroids	1902 (72.1)
Topical nonsteroids	552 (20.9)
Intralesional triamcinolone	391 (14.8)
Nontraditional treatments, or other treatments	1 (0.04)
*Patients with moderate‐to‐severe AA*	
Overall study population	1144 (100.0)
Overall study population with treatment	537 (46.9)
Topical steroids	309 (27.0)
Oral steroids	260 (22.7)
Systemic antihistamines	158 (13.8)
Topical nonsteroids	89 (7.8)
Immunomodulator	26 (2.3)
Intralesional triamcinolone	26 (2.3)
Finasteride	14 (1.2)
Phototherapy	3 (0.3)
Acupuncture	1 (0.1)
Platelet‐rich plasma	1 (0.1)

*Note*: Patients are not mutually exclusive.

Abbreviation: AA, alopecia areata.

Of note, patients were not mutually exclusive in the analysis. The different treatment types prescribed based on the disease severity are represented in Table S3.

Nearly 79.3% (*n* = 3001) of the patients with AA started treatment upon diagnosis. The average number of days between the first diagnosis and treatment initiation was 18 days, with the minimum being the same day of diagnosis and the maximum being 1623 days. Most of the patients (*n* = 2425, 80.8%) initiated treatment on the same day of diagnosis (Tables S4 and S5).

### 3.5 Specialty analysis

For the mild AA and moderate‐to‐severe AA subcohorts, the most frequently visited specialty was dermatology (mild AA subcohort: *n* = 2307, 87.4%; moderate‐to‐severe AA subcohort: *n* = 546, 47.7%). The other frequently visited specialties included general practice/family medicine (mild AA subcohort: *n* = 308, 11.7%; moderate‐to‐severe AA subcohort: *n* = 337, 29.5%), internal medicine–others (mild AA subcohort: *n* = 49, 1.9%; moderate‐to‐severe AA subcohort: *n* = 101, 8.8%), and pediatrics (mild AA subcohort: *n* = 39, 1.5%; moderate‐to‐severe AA subcohort: *n* = 76, 6.6%) (Table 3). The evaluation of specialties consulted showed that during the post‐index period, 75.76% (*n* = 2866) of patients with AA visited dermatologists. Nearly 96.4% of these patients visited the dermatologist on the same day of diagnosis; the average time to visit the dermatologist was 4 days (Table S6).

**TABLE 3:** Specialty visited (from index date to the 12‐month post‐index period).

Patients, *N* (%)	
*Patients with mild AA*	
Overall study population in the index period	2639 (100)
Dermatology	2307 (87.4)
General practice/family medicine	308 (11.7)
IM—others	49 (1.9)
Pediatrics	39 (1.5)
Obstetrics/gynecology	4 (0.2)
IM—rheumatology	3 (0.1)
IM—critical care/intensivist	1 (0.0)
Paramedic/allied healthcare	36 (1.4)
IM—endocrinology	4 (0.2)
*Patients with moderate‐to‐severe AA*	
Overall study population in the index period	1144 (100.0)
Dermatology	546 (47.7)
General practice/family medicine	337 (29.5)
IM—others	101 (8.8)
Pediatrics	76 (6.6)
Obstetrics/gynecology	67 (5.9)
IM—rheumatology	29 (2.5)
IM—critical care/intensivist	23 (2.0)
Paramedic/allied healthcare	21 (1.8)
IM—endocrinology	12 (1.0)
*Average time to see dermatologist from index date to end of study period*	
Number of patients with AA diagnosis	3783 (100)
Number of patients with AA diagnosis and treatment	2866 (75.76)
Average time number of days to visit dermatologist	4
Median number of days to visit dermatologist	0
Standard deviation	49
Minimum number of days to visit dermatologist	0
Maximum number of days to visit dermatologist	1580

*Note*: Patients are not mutually exclusive.

Abbreviations: AA, alopecia areata; IM, internal medicine.

### 3.6 Healthcare resource utilization

#### 3.6.1 HCRU and costs of associated comorbidities in patients with AA

##### Overall HCRU of associated comorbidities in patients with AA

Of the total 11 851 patients, comorbidity data were available for 9309 patients (78.55%) during the post‐index period. Overall, HCRU and associated costs for patients in the comorbidity groups (autoimmune and Th2‐mediated immune disorders: 7025 patients; psychological disorders: 226 patients; other comorbidities: 4440 patients) were analyzed during the 12‐month post‐index period.

All‐cause median gross costs were highest for patients with psychological disorders (US $1938.26 [$36.39–$110 699.67]).

For disease‐specific costs, the median gross cost in patients with psychological disorders was US $224.99 ($2.46–$42 578.67), in patients with autoimmune and Th2‐mediated immune disorders was US $103.70 ($1.16–$51 024.24) and in patients with other comorbidities was US $307.79 ($1.08–$51 468.93). The patients with other comorbidities contributed the most toward the disease burden (35%) (Table S7).

##### All‐cause HCRU and cost of associated comorbidities in patients with AA: by visit type

For inpatient visits, the median gross cost reported was highest for patients with AA with other comorbidities (US $4101.19 [$192.00–$51 468.93]). The cost incurred for outpatient and ED visits was maximum for patients with AA with psychological disorders (US $1811.72 [$36.39–$94 254.35] and US $255.92 [$11.29–$5215.22], respectively) (Table 4).

**TABLE 4:** Healthcare resource utilization and costs for associated comorbidities in patients with AA by visit type (from index date to the 12‐month post‐index period).

Population cohort	Analysis of claims								Analysis of cost				
	Patients, *N* (%)	Total	Mean	SD	Median	Minimum	Maximum	Total gross cost (US$)	Mean	SD	Median	Minimum	Maximum
Overall study population in the index period, *N* = 11 851 (100%)													
Overall patients with comorbidities in the 12‐month post‐index period (follow‐up), *N* = 9309 (78.55%)													
All‐cause													
Autoimmune and Th2‐mediated immune disorders													
Inpatient	239	300	1.3	0.59	1	1	5	1 178 032.39	4929.01	5503.90	3327.05	4.44	44 666.65
Outpatient	7024	79 711	11.3	10.59	8	1	110	8 275 198.32	1178.13	2063.43	513.95	3.23	45 901.70
Emergency department	818	2121	2.6	3.11	2	1	43	312 002.72	381.42	507.79	212.43	9.53	5242.44
Psychological disorders													
Inpatient	26	37	1.4	1.21	1	1	7	173 134.98	6659.04	8617.38	4095.16	572.27	42 578.67
Outpatient	226	4578	20.3	19.97	15	1	116	817 743.42	3618.33	7493.03	1811.72	36.39	94 254.35
Emergency department	62	163	2.6	2.74	2	2	14	32 043.33	516.83	796.25	255.92	11.29	5215.22
Other comorbidities													
Inpatient	211	300	1.4	1.24	1	1	17	1 371 292.51	6499.02	7369.32	4101.19	192.00	51 468.93
Outpatient	4437	57 975	13.1	11.88	9	1	117	6 918 666.57	1559.31	2364.48	815.89	13.36	42 031.40
Emergency department	513	1306	2.5	3.50	2	1	52	213 149.92	415.49	606.03	223.51	9.53	5796.98
Disease‐specific													
Autoimmune and Th2‐mediated immune disorders													
Inpatient	21	24	1.1	1.0	0.7	1	4	137 785.85	6561.23	9992.68	2485.16	643.32	37 244.56
Outpatient	6994	21 853	3.1	2.0	3.2	1	44	1 964 757.57	280.92	1039.89	102.09	1.16	36 947.50
Emergency department	100	160	1.6	1.0	1.1	1	9	26 175.52	261.76	273.51	188.81	11.72	1425.43
Psychological disorders													
Inpatient	5	11	2.2	1.0	2.7	1	7	49 911.86	9982.37	18 261.07	2383.81	572.27	42 578.67
Outpatient	215	829	3.9	2.0	5.2	1	28	97 059.93	451.44	732.72	207.45	2.46	4475.37
Emergency department	10	12	1.2	1.0	0.4	1	2	2496.21	249.62	247.34	157.01	52.35	794.77
Other comorbidities													
Inpatient	86	121	1.4	1.0	1.8	1	17	671 096.53	7803.45	9230.51	4673.53	348.37	51 468.93
Outpatient	4392	19 247	4.4	3.0	4.7	1	52	2 850 145.74	648.94	1150.47	300.90	1.08	26 210.17
Emergency department	84	167	2.0	1.0	1.7	1	9	32 473.35	386.59	430.26	232.90	11.46	1957.04

*Note*: Conversion factor: 1 Emirati Dirhams = 0.272 US$.

Abbreviations: AA, Alopecia areata; SD, standard deviation; Th2, T‐helper 2.

##### All‐cause HCRU and cost of associated comorbidities in patients with AA: by activity type

The median net cost incurred for medications (US $270.83 [$0.63–$82 971.13]), services (US $454.38 [$11.97–$14 886.45]), and procedures (US $907.37 [$6.75–$17 166.19]) was the highest for patients with psychological disorders. For consumables and DRG, the cost was highest for patients with other comorbidities (US $158.11 [$0.27–$11 050.94] and US $8250.86 [$1101.03–$39 706.05], respectively) (Table 5).

**TABLE 5:** HCRU and costs for associated comorbidities in patients with AA by activity type (from index date to the 12‐month post‐index period).

Population cohort	Analysis of claims							Analysis of cost					
	Patients, *N* (%)	Total	Mean	SD	Median	Minimum	Maximum	Total net cost (US$)	Mean	SD	Median	Minimum	Maximum
Overall study population in the index period, *N* = 11 851 (100%)													
Overall patients with comorbidities in the 12‐month post‐index period (follow‐up), *N* = 9309 (78.55%)													
All‐cause													
Autoimmune and Th2‐mediated immune disorders													
Medications	6471	34 272	5.3	4.0	4.9	1	89	2 061 396.83	318.56	1060.93	111.35	0.30	33 508.19
Procedure	5859	28 057	4.8	3.0	5.4	1	65	3 980 645.85	679.40	1336.37	241.68	1.74	19 456.18
Consumables	487	799	1.6	1.0	2.1	1	26	200 300.56	411.29	814.68	151.29	0.27	7157.16
Services	6758	37 613	5.6	4.0	4.8	1	64	2 123 900.99	314.29	656.04	134.27	1.36	28 385.77
DRG	32	36	1.1	1.0	0.4	1	3	181 329.12	5666.54	4479.06	3696.64	1111.33	19 943.51
Psychological disorders													
Medications	213	1773	8.3	6.0	8.3	1	52	277 881.92	1304.60	6143.14	270.83	0.63	83 124.12
Procedure	211	1898	9.0	6.0	10.3	1	57	391 109.39	1853.59	2561.01	909.04	6.76	17 197.84
Consumables	28	62	2.2	1.0	2.5	1	13	15 543.04	555.12	849.91	133.55	2.53	3052.95
Services	221	1979	9.0	7.0	8.4	1	55	188 791.97	854.27	1517.28	454.38	11.99	14 913.90
DRG	8	8	1.0	1.0	0.0	1	1	52 577.80	6572.23	5730.75	4146.28	1734.40	18 687.81
Other comorbidities													
Medications	4014	23 466	5.8	4.0	5.7	1	81	1 792 217.72	446.48	1170.34	173.42	0.27	32 252.56
Procedure	4166	23 490	5.6	4.0	6.0	1	74	3 722 997.02	893.67	1605.95	384.22	1.63	27 821.55
Consumables	394	718	1.8	1.0	3.4	1	56	208 362.15	528.83	1144.18	158.40	0.27	11 059.41
Services	4299	26 083	6.1	4.0	5.4	1	64	1 599 745.62	372.12	1107.83	139.93	2.72	51 976.72
DRG	30	38	1.3	1.0	0.5	1	3	243 889.01	8129.63	8250.86	6053.76	1103.06	39 779.26
Disease‐specific													
Autoimmune and Th2‐mediated immune disorders													
Medications	5221	10 097	1.9	1.0	1.7	1	23	2 551 518	133.25	954.07	25.35	0.29	29 573.83
Procedure	2977	5202	1.7	1.0	1.6	1	26	2 432, 864.84	222.69	572.59	89.65	1.74	15 216.83
Consumables	50	63	1.3	1.0	0.7	1	5	49 752.07	271.14	1073.97	34.28	0.27	7159.62
Services	5771	11 089	1.9	1.0	1.6	1	19	1 987 409.08	93.84	226.38	52.97	1.36	9332.94
DRG	2	2	1.0	1.0	0.0	1	1	10 001.00	1362.61	353.94	1362.61	1112.33	1612.88
Psychological disorders													
Medications	138	370	2.7	2.0	2.7	1	12	41 476.11	300.56	1245.34	72.51	0.56	14 245.01
Procedure	95	266	2.8	1.0	4.4	1	28	53 548.04	563.66	1183.33	276.13	9.81	10 301.93
Consumables	6	11	1.8	1.0	2.0	1	6	2633.16	438.86	900.61	90.20	2.43	2272.74
Services	144	285	2.0	1.0	1.9	1	10	41 540.81	288.48	1249.99	94.87	11.73	14 779.00
DRG	–	–	–	–	–	–	–	–	–	–	–	–	–
Other comorbidities													
Medications	2546	7155	2.8	2.0	2.8	1	56	879 595.10	345.49	815.74	100.99	0.29	15 482.54
Procedure	3732	8804	2.4	2.0	2.3	1	56	1 636 396.32	438.48	996.57	207.98	1.50	25 986.89
Consumables	104	240	2.3	1.0	5.9	1	56	79 798.21	767.29	1664.30	107.32	0.27	10 789.16
Services	3816	8897	2.3	2.0	2.2	1	31	578 415.64	151.58	1027.83	52.27	1.36	51 468.93
DRG	8	8	1.0	1.0	0.0	1	1	80 636.63	10 079.58	7165.62	7565.38	1886.48	20 461.43

*Note*: Conversion factor: 1 Emirati Dirhams = 0.272 US$.

Abbreviations: AA, Alopecia areata; DRG, diagnosis‐related group; HCRU, healthcare resource utilization; SD, standard deviation; Th2, T‐helper 2.

##### Disease‐specific HCRU of associated comorbidities in patients with AA: by visit type

The median gross cost reported for inpatient visits was US $2383.81 ($572.27–$42 578.67) in patients with psychological disorders, US $2485.16 ($643.32–$37 244.56) in patients with autoimmune and Th2‐mediated immune disorders, and US $4673.53 ($348.37–$51 468.93) in patients with other comorbidities. For outpatient visits, cost reported was US $207.45 ($2.46–$4475.37) in patients with psychological disorders, US $102.09 ($1.16–$36 947.50) in patients with autoimmune and Th2‐mediated immune disorders, and US $300.90 ($1.08–$26 210.17) in patients with other comorbidities. The cost for ED visits in patients with psychological disorders was US $157.01 ($52.35–$794.77), in patients with autoimmune and Th2‐mediated immune disorders was US $188.81 ($11.72–$1425.43), and in patients with other comorbidities was US $232.90 ($11.46–$1957.04) (Table 4).

##### Disease‐specific HCRU of associated comorbidities in patients with AA: by activity type

For patients with autoimmune and Th2‐mediated immune disorders, the cost was US $25.35 ($0.29–$29 573.83) for medications, US $89.65 ($1.74–$15 216.83) for procedures, US $52.97 ($1.36–$9332.94) for services, US $34.28 ($0.27–$7159.62) for consumables, and US $1362.61 ($1112.33–$1612.88) for DRG. For patients with psychological disorders the cost was US $72.51 ($0.56–$14 245.01) for medications, US $276.13 ($9.81–$10 301.93) for procedures, US $94.87 ($11.73–$14 779.00) for services, and US $90.20 ($2.43–$2272.74) for consumables. The median net cost incurred by patients with other comorbidities was US $100.99 ($0.29–$15 482.54) for medications, US $207.98 ($1.50–$25 986.89) for procedures, US $52.27 ($1.36–$51 468.93) for services, US $107.32 ($0.27–$10 789.16) for consumables, and US $7565.38 ($1886.48–$20 461.43) for DRG (Table 5).

## 4 DISCUSSION

AA is an autoimmune disease with a multifaceted impact on the lives of affected individuals. It hampers QOL while imposing a high economic and societal burden.^9^, ^12^, ^26^ Although AA is associated with a considerable disease burden, data on the epidemiology, treatment patterns, and HCRU are limited in the UAE. Therefore, a retrospective analysis using an e‐claims database was conducted to evaluate the demographics, treatment patterns, comorbidity burden, and HCRU specific to AA‐related comorbidities in patients with AA in the UAE.

The prevalence of AA in the current study was highest in the age group of those older than 18 years, with a mean age of around 36 years, in line with established literature.^2^, ^31^, ^32^ Unlike previously published studies, the present analysis reported a male predominance in the demographic characterization of AA.^32^, ^33^, ^34^ The DRWD e‐claims database used for the analysis principally includes expat populations that are predominantly male (male to female expatriate ratio: 3:1), thereby leading to a sex preponderance in the analysis of patients with AA.^35^ Male preponderance observed in our study could also be attributed to certain cultural reasons. In the UAE, growing a beard is considered very important for men and the beard area is one of the common sites affected in AA.

The association between AA and comorbid conditions, including thyroid disorders, diabetes, atopic dermatitis, psychological disorders, psoriasis, lupus erythematosus, hyperlipidemia, and nutritional deficiencies, has been explored and reported in multiple studies.^6^, ^7^, ^36^ Studies have demonstrated a bidirectional association between AA and comorbid conditions (autoimmune conditions and psychiatric conditions), suggesting a common underlying pathogenic mechanisms in these conditions.^37^, ^38^ Our analysis delineated the prevalent comorbidities as autoimmune and Th2‐mediated immune disorders (contact dermatitis, eczema, atopic dermatitis), psychiatric disorders (anxiety, depression), and other comorbidities (hyperlipidemia, hypertension, thyroid disorder), accounting for up to 78.55% of the total population during the post‐index period. A cross‐sectional study reported that 85% of respondents had one or more comorbidity, including anxiety and/or depression (47%), allergic rhinitis (35%), and thyroid disease (30%).^39^ An observational study reported a high prevalence of depression (adjusted hazard ratio [aHR], 1.30 [95% confidence interval [CI], 1.04–1.62]) and anxiety (aHR, 1.33 [95% CI, 1.09–1.63]) in patients with AA, leading to a negative impact on mental health treatment burden.^40^ In our study, the prevalence of comorbid psychiatric conditions in patients with AA was low (3%) compared with those with a comorbid autoimmune disorder (84.3%) or other comorbid conditions (47.1%). This could be attributed to the following reasons; first, complex psychosocial domains such as emotional and social functioning, which are significantly affected in patients with AA, may not be captured in claims databases using *ICD‐10* diagnosis codes; second, not all mental illnesses may be covered under a private health insurance scheme; and third, due to perceived stigma, patients may not discuss their mental health issues with healthcare practitioners.

A population‐based cohort study confirmed the high prevalence of atopic (37.2% vs 26.7%) and autoimmune (11.5% vs 7.9%) disorders in patients with AA versus patients without AA. The study revealed that patients with AA are at a high risk of atopic dermatitis, allergic rhinitis, autoimmune hypothyroidism, and systemic lupus erythematosus.^41^

Patients with AA often face stigmatization regarding clinically noticeable and negligible hair loss, leading to impaired QOL. Patients with AA reported a higher rate of unemployment, a variable disease course, and a negative impact on QOL, especially individuals with severe AA. Low scores for DLQI in mental health (45.7 ± 10.1), social functioning (45.8 ± 10.9), vitality (46.2 ± 9.8), and emotional functioning (46.9 ± 11.6) were observed in a cross‐sectional, noninterventional study, demonstrating a decrease in health‐related QOL. Nearly 32.1% of the patients confirmed that the condition had a significant negative impact on their QOL.^9^ Thus, adequate treatment intervention is required for effective and timely management of AA.^42^

Evidence from the literature suggests that topical corticosteroids are the most preferred treatment in patients with AA, regardless of severity. In a retrospective insurance claims database study, in patients with alopecia totalis and alopecia universalis (severe AA), the most prescribed treatments were injectables (29.2%) and topical (29.2%) and oral (28.5%) corticosteroids, which is similar to the prescription pattern noted in patients with moderate‐to‐severe AA in our study.^27^ In another retrospective observational claims analysis, 55.8% of patients with AA (regardless of severity) were prescribed treatments consisting primarily of topical steroids (80.3%).^25^ Similar to published studies, in the current analysis, among patients with mild severity, treatments were focused around topical steroids (72% receiving them in the first 12 months and 73% over the entire follow‐up period), whereas patients with moderate‐to‐severe AA received other types of therapies, beyond those prescribed for patients with mild AA. Although topical steroids remain the most commonly prescribed medication, only 27% of patients in the moderate‐to‐severe group received them initially. This prescription pattern demonstrates inconsistencies in what physicians are prescribing to patients with more severe cases. Of note, not all patients receive therapies, potentially because there are not effective options. However, most of these conventionally available treatments for AA, including nonspecific broad immunosuppressants such as steroids, are either off‐label or approved for short‐term use owing to associated side effects; therefore, there is a need for further research to develop targeted therapies for AA.^43^

Of late, the advent of newer treatment modalities for AA targeting the JAK–STAT pathway has provided hope for the effective management of AA with improved clinical efficacy and safety.^18^, ^43^ On June 13, 2022, the US Food and Drug Administration (FDA) approved the first systemic treatment, i.e. baricitinib, a JAK inhibitor, in the management of adult patients with AA.^44^ Another kinase inhibitor, ritlecitinib, was approved by the FDA in June 2023 for the management of adult patients with AA.^45^ According to BRAVE‐AA1 (Study of Baricitinib [LY3009104] in Participants With Severe or Very Severe Alopecia Areata), BRAVE‐AA2 (Study of Baricitinib [LY3009104] in Adults With Severe or Very Severe Alopecia Areata), and ALLEGRO 2b/3 (PF‐06651600 for the Treatment of Alopecia Areata), baricitinib and ritlecitinib provide positive outcomes in terms of hair regrowth and improve the severity of the alopecia tool score with its continued use. In BRAVE‐AA1 and BRAVE‐AA2, 17% to 22% of patients taking oral baricitinib 2 mg and 32% to 35% of patients taking baricitinib 4 mg achieved a Severity of Alopecia Tool (SALT) score of ≤20 (80% or more scalp hair coverage), compared with 3% to 5% taking placebo at week 36.^46^ The results of the ALLEGRO‐2b/3 trials in adolescents and adult patients with AA demonstrated that 23% of patients treated with ritlecitinib 50 mg had SALT ≤20 compared with 1.6% with placebo at week 24.^3^, ^47^, ^48^ While the newer therapies are available, the cost‐effectiveness of these treatments needs to be evaluated. Since the UAE is not a health technology assessment market, the health authority in the UAE would be conducting specific evaluations to determine the clinical value and cost prior to any reimbursement.

Apart from pharmacological treatments, certain nonpharmacological interventions are also being explored for AA that are both safe and effective. These include microneedling, electroacupuncture, phototherapy, and pulsed‐laser treatment. However, these approaches might be cost‐ineffective, require multiple sessions, and have the potential risk of hair loss recurrence.^49^, ^50^

Analysis of HCRU among patients with AA and comorbidities indicates noticeable costs due to autoimmune and Th2‐mediated immune disorders (contact dermatitis, eczema, atopic dermatitis), psychological disorders (depression, anxiety), and other comorbidities (hyperlipidemia, thyroid disorder, diabetes, hypertension). A high prevalence of psychiatric and autoimmune disorders in patients with AA has been reported.^7^, ^51^, ^52^ This leads to an increase in the economic burden in patients with AA, where comorbidities account for the maximum share.^26^ Furthermore, it was observed that the presence of at least one comorbidity, whether atopic, autoimmune, mental health, or cardiovascular, led to a substantial increase in cost when compared with patients without comorbidities.^53^

Interestingly, insurance is often denied for novel and advanced AA treatment, largely due to AA being regarded as a cosmetic treatment rather than a medical necessity.^54^, ^55^ Hence, nearly 33% and 15% of patients with AA were willing to pay OOP for a permanent cure and disease control, respectively, to augment their QOL.^56^ A questionnaire‐based survey conducted in the United States reported considerable OOP costs (60.1% patients), with the majority of patients indicating hair appointments (81.8%) and vitamins/supplements (67.7%) to be financially burdensome.^23^ Another cross‐sectional study conducted in Germany, Austria, and Switzerland reported a mean of €1248 per person per year as OOP cost on AA‐related necessities, including hair‐replacement products, nutritional supplements, and complementary medicines. The expense is primarily influenced by disease severity and duration, along with the preferred treatment selected.^57^ Since AA is a well‐established disorder with currently approved therapies, it should be highlighted that AA should be regarded as an autoimmune inflammatory disease and should be reimbursed like atopic dermatitis and psoriasis, rather than regarding it as a cosmetic disorder.

Despite providing substantial information on different aspects of AA in the UAE, the current analysis has certain limitations. Although claims data provide valuable statistics on healthcare outcomes, treatment patterns, resource utilization, and costs, they are not intended for research and are gathered for the purpose of reimbursement. The study population included only privately insured patients in the UAE, mainly the expatriate population. Lack of sociodemographic data among the subcohorts was also prevalent during the analysis. Thus, the findings obtained cannot be generalized to all patients with AA in the UAE. We have limitations pertinent to continuous enrollment. When the patients submit a claim in the e‐claims portal, it gets recorded in the system; however, when a claim is not submitted through the e‐claims portal but submitted outside the database, it is not recorded. In addition, the possibility of selection bias was present, since 70% of the patients had premium insurance services. Moreover, claims‐based studies often have missing or inaccurate information, leading to bias in the results. Also in the current study, we found a very high comorbidity burden especially autoimmune and other comorbidities. Of note, AA is considered a cosmetic condition in the UAE, so there may be denial of insurance coverage and perhaps even an underrepresentation of AA diagnosis codes in this database, and, consequently, this may result in more documentation of AA in patients with a more complicated health background for which they are being treated. Certain clinical and disease‐specific parameters, including the duration and severity of the disease, were not available. The possibility of misclassification of certain comorbid conditions and disease severity cannot be ruled out. The nonpharmacological/nonprescription management of AA is also not captured in the claims study. Since AA is not reimbursed in the UAE, the cost incurred by patients would be mostly OOP. The database analysis did not capture any OOP expense or patient share incurred by the patients, including any over‐the‐counter medications taken by the patient, alternative therapies not reimbursed by insurance, or other expenses at the activity level. The database only records the conditions, procedures, and treatments for the ongoing insurance plan. Hence, the results might not be a true representation of AA‐related costs in the UAE. The baseline information may be underreported owing to the availability of limited pre‐index data. The data on disease‐specific medications, HCRU, and costs are suggestive. Furthermore, one‐to‐one mapping between the diagnosis and medications/procedures/consumables is not available from the DRWD. Therefore, some of the AA‐related costs might not be associated with the disease. The severity of the disease was defined based on the type of treatment prescribed for AA, in line with a previous study.^25^ However, severity, as defined by the prescribed treatment, may not necessarily be analogous to the proportion of scalp hair loss. Since AA treatments are largely off‐label and ineffective and patients may have different treatment goals (e.g. someone with universalis may only want scalp regrowth and use injectable steroids), it may be that patients receiving topical therapies have substantial hair loss but are not willing to take systemic therapies. Therefore, defining severity by the treatment patients received can tell us something about how the physician views severity, but it is limited in interpretation as the goal of treatment is unknown. This may not accurately reflect the disease and does not account for severity in inpatients who did not receive prescription medications. Furthermore, treatment such as for those with contact immunotherapy (dinitrochlorobenzene, diphenylcyclopropenone, and squaric acid dibutyl ester) may not be listed in the database, despite being used for treatment of AA in the UAE. Contact immunotherapy would not be captured as a separate line of therapy, as the code may not be a reimbursable one. Also, there is no information on whether the medication was prescribed for AA or may have been prescribed for other conditions. In addition, there is no information available to confirm whether medications were consumed or taken as prescribed.

Depending on how these patients are distributed in usual care, the robustness of the analysis by cohort and age may be limited. If a cohort was underrepresented in the population, it might impact the cost distribution. The indirect cost pertaining to the work productivity and QOL of the patients was not evaluated as part of the economic burden in patients with AA.

Nonetheless, the current study provides an in‐depth analysis of real‐time data on patient demographics, treatment patterns, HCRU, and associated costs for patients with AA in the UAE. The study provides data on private insurance, mostly for the expatriate population (89% of the entire population).^30^

In conclusion, this comprehensive analysis provides real‐world evidence for the privately insured UAE population showing that AA is associated with a substantial clinical burden. The study highlights the high rate of comorbidities, the treatment patterns currently followed, HCRU, and the costs for patients with AA and associated comorbidities. The study findings demonstrate the most prevalent comorbid disorders, including contact dermatitis and eczema, with steroids (topical and oral) being the most frequently prescribed treatment. However, novel therapies for AA including JAK inhibitors are currently not prescribed in the UAE, as per the claims database. Patients with AA and associated comorbidities have high resource utilization attributed to inpatient visits, with costs due to procedures contributing the most.

The observed treatment patterns are consistent with international guidelines, which are primarily off‐label and inadequate to meet treatment goals. Investing in novel therapies for AA, which target the underlying autoimmune pathway and thereby aid in sustainable regrowth of hair, could provide a new and long‐term therapeutic option in the management of patients with AA.

Innovative therapies, including recently approved JAK inhibitors and kinase inhibitors with good efficacy and safety, could aid in achieving treatment goals, therefore addressing the gap in effective therapies. This allows the payers to utilize the funds more efficiently, thereby reducing resource use and cost burdens associated with currently used ineffective standard‐of‐care therapies. The study findings underscore the need to include these therapies in clinical practice in the UAE, with expanding patient access and insurance reimbursement. However, long‐term analysis throughout the treatment trajectory of AA is warranted for improved clinical outcomes and to reduce the economic burden.

## CONFLICT OF INTEREST STATEMENT

H.M.A., M.W.A.Z., and F.E. are full‐time employees of Pfizer Inc. The other authors do not report any conflicts of interest.

## Supporting information


Table S1.

## Data Availability

The data for the current study are not publicly available. The data were obtained from Dubai Health Insurance Corporation (DHIC) following all legal and ethical procedures to use the de‐identified patient data. DHIC is the owner of the DRWD. There is an agreement between IQVIA and DHIC to utilize deidentified data for health insurance claims data‐based analysis and research studies. IQVIA agreed to adhere to measures to comply with applicable laws, including those related to data protection. The summarized results for this study were made available to the authors through IQVIA (third‐party license). The deidentified data of this study were handled by IQVIA Dubai Full time employee. All data generated or analyzed during this study are included in this published article as Supporting Information files.
