# Supplementary material for: A retrospective analysis of disease epidemiology, comorbidities, treatment patterns, and healthcare resource utilization of alopecia areata in the United Arab Emirates using claims database
Source: J Dermatol. 2024 Jul 25;51(9):1157–71. doi: 10.1111/1346-8138.17381 (PMC11484132; doi:10.1111/1346-8138.17381)
Supplement: Supplementary file 1 — Table S1. [file JDE-51--s001.docx]

**SUPPLEMENTARY TABLES**

**Table S1: Alopecia areata diagnosis codes**

| **Code** | **Description** |
| --- | --- |
| **L63** | **Alopecia Areata** |
| L63.0 | Alopecia (capitis) totalis |
| L63.1 | Alopecia universalis |
| L63.2 | Ophiasis |
| L63.8 | Other alopecia areata |
| L63.9 | Alopecia areata, unspecified |

**Table S2: Excluded patients with the following ICD10 codes**

| **Code** | **Description** | **Grouping** |
| --- | --- | --- |
| F63.3 | Trichotillomania | Habit and impulse disorders |
| L64.0 | Drug-induced androgenic alopecia | Androgenic alopecia |
| L64.8 | Other androgenic alopecia | Androgenic alopecia |
| L64.9 | Androgenic alopecia, unspecified | Androgenic alopecia |
| L65.0 | Telogen effluvium | Other nonscarring hair loss |
| B35.0 | Tinea barbae and tinea capitis | Dermatophytosis |
| L66.1 | Lichen planopilaris | Cicatricial alopecia (scarring hair loss) |
| L66.3 | Perifolliculitis capitis abscedens | Cicatricial alopecia (scarring hair loss) |
| L66.4 | Folliculitis ulerythematosa reticulata | Cicatricial alopecia (scarring hair loss) |
| L66.8 | Other cicatricial alopecia | Cicatricial alopecia (scarring hair loss) |
| L66.9 | Cicatricial alopecia, unspecified | Cicatricial alopecia (scarring hair loss) |
| L65.1 | Anagen effluvium | Other nonscarring hair loss |
| L65.2 | Alopecia mucinosa | Other nonscarring hair loss |
| L65.8 | Other specified nonscarring hair loss | Other nonscarring hair loss |
| L65.9 | Nonscarring hair loss, unspecified | Other nonscarring hair loss |
| L66.0 | Pseudopelade | Cicatricial alopecia (scarring hair loss) |
| L66.2 | Folliculitis decalvans | Cicatricial alopecia (scarring hair loss) |

**Table S3: Treatment type and severity**

| **Type** | **Severity** |
| --- | --- |
| Acupuncture | Mild |
| CPT Procedure | Mild |
| Intralesional triamcinolone | Mild |
| Non-traditional treatments, or other treatments | Mild |
| Topical non-steroids | Mild |
| Topical steroids | Mild |
| Finasteride | Moderate to severe |
| Immunomodulator | Moderate to severe |
| Oral steroids | Moderate to severe |
| Phototherapy | Moderate to severe |
| Platelet rich plasma | Moderate to severe |
| Systemic antihistamines | Moderate to severe |
| Systemic non-steroids | Moderate to severe |

Abbreviation: CPT: Current procedural terminology

**Table S4: Average time between first AA diagnosis and treatment (mild or moderate-to-severe)**

|  | **Number of Patients, N (%)** |
| --- | --- |
| Number of patients AA Diagnosis | 3,783 (100) |
| Number of patients AA diagnosis and treatment | 3,001 (79.3) |
| Average time between first AA diagnosis and treatment (days) | 18 |
| Median (days) | 0 |
| Standard deviation | 95 |
| Minimum time between first AA diagnosis and treatment (days) | 0 |
| Maximum time between first AA diagnosis and treatment (days) | 1623 |

**Note: 0 - indicates patient has received treatment at the time of diagnosis**

Abbreviations: AA: Alopecia areata; N: Number of patients.

**Table S5: Diagnosis and treatment time distribution**

| **Number of Days** | **Number of Patients, N (%)** |
| --- | --- |
| Same Day | 2,425 (80.8) |
| 1–5 | 217 (7.2) |
| 6–10 | 35 (1.2) |
| 11–20 | 36 (1.2) |
| 21–30 | 44 (1.5) |
| 31–40 | 28 (0.9) |
| 41–50 | 25 (0.8) |
| 51–100 | 60 (2.0) |
| 101–200 | 53 (1.8) |
| 201–300 | 33 (1.1) |
| 301–400 | 17 (0.6) |
| 501–1000 | 10 (0.3) |
| 401–500 | 10 (0.3) |
| >1000 | 8 (0.3) |

**Table S6: Time distribution to consult dermatologists since AA diagnosis date**

| **Number of Days** | **Number of Patients, N (%)** |
| --- | --- |
| Same day | 2,762 (96.4) |
| One month | 68 (2.4) |
| Two months | 11 (0.4) |
| Three months | 5 (0.2) |
| Four months | 2 (0.1) |
| Five months | 2 (0.1) |
| Six months | 1 (0.0) |
| One year | 5 (0.2) |
| > One year | 10 (0.3) |

**Table S7: Overall HCRU and Costs of Associated Comorbidities in Patients with AA Patients**

| **Overall study population in index period N (%) = 11,851 (100)** | | | | | | | | | | | | | |
| --- | --- | --- | --- | --- | --- | --- | --- | --- | --- | --- | --- | --- | --- |
| **Overall patients with comorbidities in 12-month post-index period (follow-up) N (%) = 9,309 (78.55)** | | | | | | | | | | | | | |
| **Disease-specific** | | | | | | | | | | | | | |
| **Analysis of claims** | | | | | | | | **Gross cost (USD)** | | | | | |
| **Population Cohort** | **Patients, N (%)** | **Total** | **Mean** | **SD** | **Median** | **Min** | **Max** | **Total cost** | **Mean** | **SD** | **Median** | **Min** | **Max** |
| Auto-immune and Th2-mediated immune disorders | 7,013 | 22,037 | 3.1 | 3.24 | 2 | 1 | 44 | 2,128,718.94 | 303.54 | 1,282.01 | 103.70 | 1.16 | 51,024.24 |
| Psychological Disorders | 222 | 852 | 3.8 | 5.08 | 2 | 1 | 28 | 149,468.00 | 673.28 | 2,926.05 | 224.99 | 2.46 | 42,578.67 |
| Others | 4,427 | 19,535 | 4.4 | 4.82 | 3 | 1 | 56 | 3,553,715.62 | 802.74 | 2,173.77 | 307.79 | 1.08 | 51,468.93 |
| **All-cause** | | | | | | | | | | | | | |
| Auto-immune and Th2- mediated immune disorders | 7,025 | 82,132 | 11.7 | 11.02 | 8 | 1 | 119 | 9,765,233.44 | 1,390.07 | 2,762.00 | 545.45 | 3.23 | 60,432.40 |
| Psychological Disorders | 226 | 4,778 | 21.1 | 20.80 | 15.5 | 2 | 125 | 1,022,921.72 | 4,526.20 | 9,423.92 | 1,938.26 | 36.39 | 110,699.67 |
| Others | 4,440 | 59,581 | 13.4 | 12.38 | 10 | 1 | 117 | 8,503,109.01 | 1,915.12 | 3,504.57 | 874.83 | 13.36 | 55,117.37 |

Abbreviations: Min: Minimum; Max: Maximum; SD: Standard deviation; Th2: T-helper 2; USD: United States dollar.

Note: Conversion factor: 1 AED=0.272 USD

**APPENDIX**

**Subcohort definitions: Based on diagnosis codes and treatment pattern.**

| **Subcohorts** | **Definitions based diagnosis codes and treatment** |
| --- | --- |
| **Mild** | Patients with AA diagnosis code for other alopecia areata (L63.8) or alopecia areata, unspecified (L63.9) and those who were prescribed topical steroids,topical non-steroids, non-traditional treatments, or other treatments ,acupuncture ,intralesional triamcinolone will be categorised as having mild disease. |
| **Moderate-to-severe** | **1.** Patients with a diagnosis code for alopecia universalis (L63.1) or alopecia totalis (L63.0) or ophiasis (L63.2).  2. Patients with a diagnosis code for Other alopecia areata (L63.8) or alopecia areata, unspecified (L63.9) and those who were prescribed any immunomodulators, oral steroids, systemic non-steroids, or phototherapy,finasteride,systemic antihistamines(ebastine and fexofenadine), platelet rich plasma. |
| **Others** | The patients with a diagnosis code for AA (L63) and not captured in any of the cohorts of mild or moderate-to severe disease code |
